# Does truth matter to voters? The effects of correcting political misinformation in an Australian sample

**DOI:** 10.1098/rsos.180593

**Published:** 2018-12-19

**Authors:** Michael J. Aird, Ullrich K. H. Ecker, Briony Swire, Adam J. Berinsky, Stephan Lewandowsky

**Affiliations:** 1 School of Psychological Science, University of Western Australia, Perth, Western Australia, Australia; 2 Department of Political Science, Northeastern University, Boston, MA, USA; 3 Department of Political Science, Massachusetts Institute of Technology, Cambridge, MA, USA; 4 School of Experimental Psychology and Cabot Institute, University of Bristol, Bristol, UK

**Keywords:** misinformation, fact-checking, political attitudes, belief change, voting behaviour, misconceptions

## Abstract

In the ‘post-truth era’, political fact-checking has become an issue of considerable significance. A recent study in the context of the 2016 US election found that fact-checks of statements by Donald Trump changed participants' beliefs about those statements—regardless of whether participants supported Trump—but not their feelings towards Trump or voting intentions. However, the study balanced corrections of inaccurate statements with an equal number of affirmations of accurate statements. Therefore, the null effect of fact-checks on participants’ voting intentions and feelings may have arisen because of this artificially created balance. Moreover, Trump's statements were not contrasted with statements from an opposing politician, and Trump's perceived veracity was not measured. The present study (*N* = 370) examined the issue further, manipulating the ratio of corrections to affirmations, and using Australian politicians (and Australian participants) from both sides of the political spectrum. We hypothesized that fact-checks would correct beliefs and that fact-checks would affect voters’ support (i.e. voting intentions, feelings and perceptions of veracity), but only when corrections outnumbered affirmations. Both hypotheses were supported, suggesting that a politician's veracity does sometimes matter to voters. The effects of fact-checking were similar on both sides of the political spectrum, suggesting little motivated reasoning in the processing of fact-checks.

## 1. Introduction

Veracity is generally considered an important attribute in politicians, yet they often make incorrect or misleading statements [1]. Such statements can shape policies and beliefs for years to come. For example, during the presidency of George W. Bush, American politicians justified the 2003 invasion of Iraq by claiming that it possessed weapons of mass destruction (WMD). Despite clear evidence to the contrary emerging after the invasion [2,3], for years many Americans continued to believe that Iraq had WMD immediately prior to the conflict [4,5]. Furthermore, a month after the release of a key US intelligence report confirming Iraq had not possessed WMD prior to the invasion [2], President Bush won re-election.

The prevalence of political misinformation undermines the public's capacity to make informed political choices [6,7]. In response to this concern, fact-checking has become increasingly common in recent years [8,9]. However, research has yet to adequately address two key questions: (1) How effectively does fact-checking a politician's statements change belief in the truth of those statements? (2) How does recurrent evidence that a politician made false statements affect support for that politician? The present study was designed to help address these questions. It was run in Australia, rather than the USA, in order to contrast misinformation from two contemporary politicians—one on the political right, one on the political left—who are otherwise comparable across many dimensions. To foreshadow, this is also important because our results suggest that the impact of politicians' false statements on the level of support they receive may differ between the USA and Australia.

The next two sections outline relevant prior literature. First, we discuss how corrective information affects beliefs, and how attitudes may impact this process. Second, we discuss how changes in attitudes and intentions may or may not follow from any change in belief. We define attitudes as generic viewpoints regarding specific issues, institutions or groups that are integral to a person's social identity [10,11]. In the specific context of this study, we will investigate partisan attitudes in particular, that is, participants' political worldview on a left–right dimension and their attitude towards the major left- versus right-leaning political parties in Australia.

## 2. Changing beliefs

Misinformation that has been corrected often continues to affect people's memories, beliefs and inferential reasoning, even if those people remember the correction and believe it to be accurate [12–17]. For example, Ecker *et al.* [18] presented participants with a fictitious news report about a robbery at a liquor store. The report first stated that police suspected the perpetrators were Aboriginal Australians, but later retracted this information, clarifying that police no longer suspected the robbers were Aboriginal. However, participants continued to rely on the corrected misinformation in answering inference questions. For example, some participants referred to the robbers speaking an Aboriginal language (which was not mentioned in the report) when asked why the shop owner had difficulties understanding the attackers. This reliance on corrected information occurred despite most participants recalling the correction when queried about it directly. In other words, corrections will often reduce but not eliminate the influence of misinformation on reasoning. This phenomenon holds for both political and non-political topics (see [19–21] for reviews).

This continued influence of misinformation despite corrections is often strongest when that misinformation is congruent with a person's pre-existing attitudes or worldview, whereas the correction is not [22]. Nyhan & Reifler [5] provided participants with mock news articles that included a misleading claim, for instance, that tax cuts under President Bush had increased government revenue or that Bush had banned stem cell research. In some conditions, the articles also included a correction of the misleading claim. These corrections were generally less effective when they were incongruent with a participant's attitudes, such as when left-wing participants were informed that Bush had not banned stem cell research. In fact, in the most right-wing participants, reading that Bush's tax cuts had not actually increased government revenue ironically resulted in *stronger* beliefs that revenue had increased, an effect known as the ‘worldview backfire effect’.

Other studies have likewise found that existing attitudes have an impact on the effectiveness of corrections [23–26]. One explanation for these attitude effects is that attitude-incongruent corrections induce motivated reasoning—the processing of new information such that existing attitudes are able to be maintained [27,28]. Proposed mechanisms of motivated reasoning include generating counterarguments to attitude-incongruent messages [29–32], bringing to mind reasons for holding one's initial attitude [28], and derogating sources of attitude-incongruent messages [7,33].

However, the strength and even presence of attitude effects appear to vary. For example, in Ecker *et al*.'s study [18], participants' levels of prejudice towards Aboriginal Australians had no impact on the effectiveness of corrections. While highly prejudiced participants referred more often to the presumed aboriginality of the robbers in general, both high- and low-prejudice groups reduced their reliance on the race-related information to a similar extent when provided with a correction (i.e. there was a main effect of racial prejudice but no interaction between prejudice and the correction). Ecker *et al*. suggested that pre-existing attitudes may have no impact on the effectiveness of a correction if belief change does not require attitude change. That is, a highly prejudiced person could accept that a particular robbery was not committed by Aboriginal people while still believing most robberies are. They could thus change their belief regarding the specific robbery without having to change their attitudes towards Aboriginal people.

By contrast, updating more general beliefs—for example, regarding the overall crime rate among Aboriginal Australians—may require some degree of attitude change. Thus, attitudes would be expected to have a greater impact on correction effectiveness with such general assertions. Ecker & Ang [23] found support for this notion in a study investigating political misinformation. They contrasted correction of a specific case of alleged misconduct with a correction of a more general case of misconduct; political attitudes were found to have little impact on retraction processing if the retraction concerned a specific case of an individual politician allegedly involved in misconduct. However, attitudes had a significant impact if the retraction concerned a general assertion that politicians from a specific party were more likely involved in misconduct.

Likewise, other studies have suggested that the processing of corrective information is affected by attitudes only if (a) the information directly challenges those attitudes and (b) those attitudes are strongly held and important to the individual [5,32,34–38]. Thus, it may be that the motivated rejection of corrections only occurs when changing beliefs would require changing strong attitudes. By contrast, when attitudes are weaker or can be maintained regardless of any belief change triggered by an event-specific correction, the efficacy of corrections may be unaffected by their attitudinal status [18,23]. In the following, we will refer to this argument—that motivated rejection of corrections occurs only when strong attitudes are directly challenged—as the ‘attitude-protection hypothesis’.^1^

Swire *et al.* [39] found results which appear consistent with this hypothesis. In their study, conducted during the 2016 US Presidential election campaign, participants were shown statements that had been made by Donald Trump on the campaign trail. Four of these statements were accurate and four were inaccurate. Initial belief in these statements was higher for Trump's supporters than non-supporters. However, fact-checks of these statements were similarly effective for both supporters and non-supporters, increasing belief in accurate statements and decreasing belief in inaccurate statements.

Swire *et al*. [39] suggested the lack of attitude effects on beliefs may have resulted from at least some of the statements being tangential to participants' core attitudes. Even if supporters and non-supporters had strong attitudes regarding Trump, those core attitudes need not have constrained people's views on the particular issues addressed in Trump's statements, such as the cost of the Iraq War. It is known that views on specific issues do not consistently map onto party preferences [40–44]. Thus, people may be able to update their belief in a politician's statements without having to change any strong attitudes. Under such conditions, the attitude-protection hypothesis would predict no attitude effects on the degree of belief change, just as Swire *et al*. observed.

Finally, there is a debate over whether, when such attitude-protective effects occur, they are stronger among right-wing individuals than left-wing individuals. There is some evidence that conservatives employ more motivated reasoning in the face of information that challenges their attitudes due to conservatives' reportedly higher levels of dogmatism, need for closure, sensitivity to threat and ‘bullshit receptivity’ [9,23,43,45–53]. Alternatively, it may be that the underlying mechanisms of motivated reasoning are the same regardless of political orientation, and that motivated reasoning therefore occurs symmetrically across political orientations [27,30,54–56]. For example, liberals and conservatives have been found to be equally likely to engage in cognitive shortcuts to render an interpretation of scientific data consonant with their attitudes [57].

The first aim of the present study was to extend Swire *et al*.'s [39] finding that general political attitudes did not moderate belief change following fact-checks of a politician's statements. Moreover, we used both a left-wing and a right-wing politician, allowing further investigation of whether attitudes impact belief change differently (a) among left-wing versus right-wing participants and (b) for statements from preferred versus non-preferred politicians.

We now turn to prior literature relevant to the second question motivating this study: How does evidence that a politician made false statements affect attitudes and voting intentions regarding that politician?

## 3. Changing attitudes and intentions

Attitudes and behavioural intentions are often more consequential, and thus more important to change, than specific beliefs (see [4,58]). To illustrate with an extreme case, if fact-checking led every citizen of a country to recognize that every statement a given politician made was false, but that politician was still liked and won election, the fact-checking would arguably be of limited value. Unfortunately, it cannot be assumed that changes in beliefs flow directly into changes in attitudes and intentions. For example, in Swire *et al*.'s [39] study, although refuting four of Trump's statements decreased belief in those statements, it had no impact on participants' feelings towards Trump or their intentions to vote for him.

The authors suggested two possible explanations of these findings. First, voters may not particularly care if politicians lack veracity. Alternatively, given that Swire *et al*.'s [39] participants were shown as many accurate as inaccurate statements, it may be that a detrimental effect of exposing Trump's inaccurate statements on voting intentions and feelings of his supporters was counteracted by a positive effect of affirming his accurate statements. Swire *et al*. thus called for further research to vary the ratio of true and false claims in order to investigate the effects of presenting more corrections than affirmations of a politician's statements. Indeed, a prior study suggests that negative information about a supported political candidate may not cause more negative attitudes towards them unless the amount of negative information reaches a sufficient magnitude [26].

However, a third explanation of these findings is also possible. It may be that voting intentions and feelings did not change because perceptions of Trump's general *tendency* to be accurate did not change. It may be that Trump's supporters would have adjusted their attitudes towards him, had they adjusted their perceptions of his general veracity, but that four correct and four incorrect statements were insufficient evidence of a deviation from their expectations. It would therefore be valuable to measure the perceptions of a politician's veracity, to distinguish whether people (a) are indeed changing their perceptions of veracity yet not changing their feelings or intentions, or (b) are not changing their perceptions of a politician's veracity in the first place.

The second aim of the present study was therefore to examine whether the balance of true and false statements from a politician might affect people's level of support. We therefore also manipulated the ratio of true and false statements presented to participants.

## 4. The present study

The present study's design largely followed that of Swire *et al*. [39]. Participants were shown real statements politicians had made, followed by fact-checks of these statements. We investigated how these fact-checks affected belief in the statements and voting intentions and feelings regarding the politicians. There were three key differences. First, we recruited Australian participants and used Australian politicians from each side of the political spectrum—namely, Bill Shorten and Malcolm Turnbull, who at the time of the study was conducted were leaders of the left-wing Labor party and right-wing Liberal party, respectively. This allowed us to test the generalizability of Swire *et al*.'s findings in a different national and cultural context. (Turnbull and Shorten seemed particularly well suited for a comparison as they were similarly unpopular with Australian voters and were also perceived as similar characters, occasionally even being referred to in the Australian media as ‘terrible twins’.) Second, we manipulated the ratio of true to false statements. Some participants received an equal number of inaccurate statements (hereafter referred to as *myths*) and accurate statements (hereafter, *facts*), whereas others received mainly myths. Third, we asked participants how often they considered the politicians to be accurate in their statements in general—that is, we measured perceptions of veracity.

Our first hypothesis (H1) was that fact-checks would increase fact beliefs and decrease myth beliefs. Associated with H1 were three subordinate research questions. We expected that (a) pre-fact-check, participants would believe more in myths from a favoured, attitude-congruent source than a non-favoured, attitude-incongruent source. (b) We were curious whether fact-check efficacy would be influenced by the congruence between information source and personal attitude, and (c) if such attitude effects were to occur, whether they would be stronger among right-wing participants or symmetrical across political orientations. Finally, our second main hypothesis (H2) was that candidate's support (voting intentions, feelings, perceived veracity) would not change when an equal number of facts and myths were fact-checked, but would decrease when participants received mostly myth corrections.

## 5. Material and methods

This study had a 2 × 2 × 2 × 3 between–within design. The within-subjects factor was fact-check (pre, post). The between-subjects factors were politician (Shorten, Turnbull), myth:fact ratio (4 : 1, 4 : 4), and participants' political orientation (left-wing, right-wing). The study was run as an experimental survey using the Qualtrics software (Qualtrics, Provo, UT).

### 5.1. Participants

The sample comprised 455 participants: 100 undergraduate students from the University of Western Australia and 355 online participants who were residents of Australia. As most participants were recruited online, various quality-control items were included in the survey; these are described in detail in the Materials section. Based on *a priori* criteria, participants were excluded if they failed an attention filter (*n* = 47), indicated that they had already completed the experiment on a different platform (*n* = 7), indicated that their data should be discarded because they had not been paying attention (*n* = 1), or failed at least one of two questions assessing basic Australian political knowledge (*n* = 42); these questions were designed to be extremely easy for Australian residents to answer correctly, and were thus intended to allow for the exclusion of participants who lacked a bare minimum of relevant knowledge, such as newly arrived international students. As some participants met more than one exclusion criterion, the final sample size was *N* = 370.

The final sample consisted of 82 undergraduates (57 female, 25 male; age range 17–45 years, *M*_age_ = 21.24, s.d._age_ = 5.70) and 288 online participants (125 female, 161 male, two undisclosed gender; age range 18–81 years, *M*_age_ = 39.23, s.d._age_ = 16.82). Online participants were recruited from multiple platforms to achieve timely completion.^2^ The undergraduates participated in exchange for course credit, while the online participants received a small reimbursement. All participants participated voluntarily after reading an ethically approved information sheet and providing informed consent. Data were collected between April and July 2017.

Participants were randomly allocated to politician and myth:fact ratio conditions; allocation to either the Shorten or the Turnbull condition determined which politician a participant saw statements from and answered questions about. Participants were split up into left-wing and right-wing groups based on their responses to a political orientation questionnaire.

### 5.2. Materials

Political orientation was assessed using a six-item scale. The scale included the five items of Ecker and Ang's [23] party-preference scale, a modification of Mehrabian's [59] conservatism–liberalism scale for use in Australia. This questionnaire requires participants to respond to five statements on a 5-point Likert scale, ranging from ‘Strongly disagree’ (1) to ‘Strongly agree’ (5). A representative item is ‘The major national media are too protective of the Labor party for my taste’. The sixth item asked participants to ‘indicate the extent to which you identify as politically left-wing or right-wing’ on a 7-point Likert scale, ranging from ‘Very left-wing’ (1) to ‘Very right-wing’ (7).

Australian political knowledge was assessed using two multiple-choice questions: ‘Who is the leader of the political party One Nation?’ and ‘Which of these people is a former Australian Prime Minister?’ In addition to the correct response options ‘Pauline Hanson’ and ‘Kevin Rudd’, respectively, each of these questions had seven lure options, which were figures from modern or historical Australian politics.

Three measures assessed various aspects of participants' views regarding the politician they were allocated. Voting intentions were assessed with the question ‘If today was election day, and you could vote directly for a new prime minister, how likely would you be to vote for Bill Shorten/Malcolm Turnbull?’ (which name a given participant was shown was determined by which politician condition that participant had been allocated to). Responses were provided on an 11-point Likert scale from ‘Extremely unlikely’ (0) to ‘Extremely likely’ (10). Feelings towards the allocated politician were assessed with a ‘feeling thermometer’, on which participants rated how favourably they felt towards the politician on a scale from 0 to 100, with higher scores indicating more favourable feelings. The perceived veracity of the allocated politician was assessed by the question ‘On the whole, how often would you say Bill Shorten/Malcolm Turnbull is accurate in what he says?’, responded to on an 11-point Likert scale from ‘Never’ (0) to ‘Always’ (10).

We compiled four inaccurate statements (myths) and four accurate statements (facts) made publically by each politician in the time period 2014–2016. An example myth was ‘Bill Shorten said nine out of ten Australians spend more than 90 minutes a day travelling to and from work’. An example fact was ‘Bill Shorten said in late 2014 that the youth unemployment rate was at a 13-year high’. Belief in each statement was assessed with the question: ‘On a scale of 0–10, do you believe Shorten's/Turnbull's statement to be true?’ responded to on an 11-point Likert scale from ‘Definitely false’ (0) to ‘Definitely true’ (10).

Additionally, a fact-check of between 41 and 69 words was generated for each statement. The fact-check associated with the abovementioned myth was ‘Shorten's statement is incorrect and misleading. Data from the Household, Income and Labour Dynamics in Australia survey show that less than 2 in 10 Australians spend more than 90 min a day travelling to and from work. The average time spent commuting was around half that time.’ The fact-check associated with the abovementioned fact was ‘Shorten's statement is correct. The Australian Bureau of Statistics report that in October 2014, the youth unemployment rate was at 13.8%. The last time it had been as high as 13.8% was in November 2001, 13 years prior to Shorten's statement.’ The full set of myths, facts and fact-checks, as well as the full set of questions can be found in the electronic supplementary material.

### 5.3. Procedure

The undergraduate participants were tested individually and completed the experiment on a computer in a laboratory. The remaining participants completed the experiment online. Participants first answered questions about their age and gender, followed by the six political orientation questions and the two political knowledge questions. Participants then responded to a first round of voting intentions, feelings and perceived veracity measures about the politician they were allocated.

Participants were told they would receive between five and eight statements from this politician. Half received four myths and four facts from their allocated politician, while the other half received four myths but only one fact (which was randomly selected from the pool of four). In total, the set of five or eight statements was presented three times; statements were always shown one at a time, in random order. On the first presentation, participants rated their belief in each statement. On the second presentation, each statement was accompanied by the appropriate fact-check. Participants could only click past these fact-checks after a minimum of 7 s had elapsed. On the third and final presentation, participants re-rated their belief in each statement.

Participants then responded to a second round of the voting intentions, feelings and perceived veracity measures. For online participants, this was followed by an educational attainment question, an attention filter, and a question regarding prior completion. Finally, all participants answered a question regarding whether they had paid attention. The median time taken to complete the survey was 8 min (the minimum time required was determined *a priori* to be 3 min; no one completed the survey faster).

## 6. Results

### 6.1. Coding

A political orientation score was calculated by averaging the responses to the six political orientation items and transforming the resulting mean onto a 0–1 scale. (All dependent variables were likewise transformed to a 0–1 scale to facilitate interpretation of scores and results.) This composite scale was associated with Cronbach's *α* = 0.92. The mean political orientation score was *M* = 0.44, s.d. = 0.26, with higher scores indicating more right-wing orientation; the sample was thus slightly left-leaning on average. Participants were split into two political orientation groups using the midpoint of the scale (excluding the actual midpoint itself): left-wing (*n* = 219; political orientation score *M* = 0.26, s.d. = 0.14) and right-wing (*n* = 128; *M* = 0.73, s.d. = 0.14). Pre- and post-fact-check myth/fact belief scores were obtained by averaging participants' pre- and post-fact-check myth/fact belief ratings, respectively. The three dependent variables of voting intentions, feelings and perceived veracity were highly correlated (*r*s > 0.76 at time 1 and *r*s > 0.67 at time 2) and were combined in a composite ‘support’ score for the main analysis, following an *a priori* analysis plan. Moreover, the politician factor (Shorten versus Turnbull) was recoded to a source congruence factor (congruent versus incongruent) that reflected the congruence between participant's attitude and the politician's affiliation.

For the sake of clarity, analyses reported below do not reflect the full experimental design; specifically, the myth:fact ratio factor was primarily of interest in the analysis of the support measure and was thus omitted from the analysis of belief measures. Likewise, the political orientation factor was only included in the analyses of belief measures, but omitted from the analysis of the support measure. Full analyses yielded identical conclusions and are provided in the electronic supplementary material.

### 6.2. Myth belief

**Figure 1.**
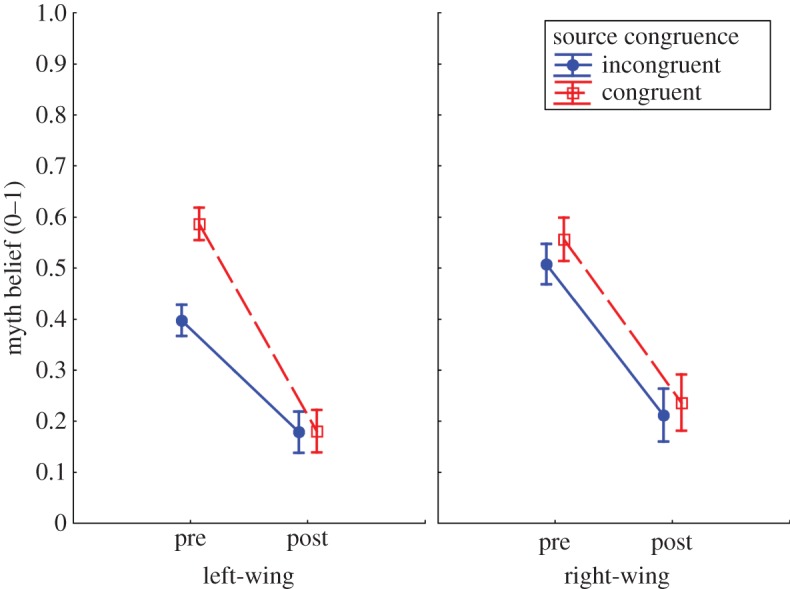
Pre- and post-fact-check myth belief across source congruence and political orientation conditions. Error bars indicate 95% confidence intervals.

To investigate the effects of fact-checks on myth belief, and how these effects might differ depending on source congruence and political orientation, we ran a 2 × 2 × 2 between–within ANOVA on myth belief scores. Fact-check (pre, post) was a within-subjects factor, and between-subjects factors were source congruence (congruent, incongruent) and political orientation (left-wing, right-wing). Table 1 shows the ANOVA results, and figure 1 shows the myth belief scores across conditions.^3^

**Table 1.** ANOVAs with myth belief and fact belief as the dependent variables. SC, source congruence; PO, political orientation; FC, fact-check.

	myth belief			fact belief		
effects	*F*_1,343_	*p*-value	$\eta_{p}^{2}$	*F*_1,343_	*p-*value	$\eta_{p}^{2}$
SC	14.87	<0.001	0.04	24.05	<0.001	0.32
PO	6.04	0.014	0.02	1.62	0.204	<0.01
SC × PO	2.95	0.087	0.01	2.81	0.095	0.01
FC	566.70	<0.001	0.62	158.03	<0.001	0.32
FC × SC	16.51	<0.001	0.05	<1		
FC × PO	<1			2.21	0.138	0.01
FC × SC × PO	9.77	0.002	0.03	3.22	0.074	0.01

This showed a main effect of fact-check, showing that myth belief was reduced from pre to post, a main effect of political orientation, showing that myth belief was somewhat greater in right-wing participants in general, as well as a main effect of source congruence, indicating that participants tended to believe more in source-congruent myths. The main effects were qualified by two interactions: the fact-check × source congruence interaction indicated that myth beliefs decreased more if the myth came from a favoured source; the three-way interaction showed that this was true primarily for left-wing participants. This indicates that left-wing participants reduced their belief in myths from a congruent source much more than their belief in myths from an incongruent source (*post hoc* interaction contrast: *F*_1,343_ = 35.10, *p* < 0.001). This effect was driven entirely by strong pre-correction belief differences (*post hoc* contrast: *F*_1,343_ = 70.83, *p* < 0.001), with particularly low baseline belief in incongruent (i.e. Turnbull) myths in left-wing participants. Thus, while left-wing participants reduced their belief in both politicians' myths to a very similar post-correction level, their beliefs had ‘further to fall’ for congruent (Shorten) myths than for incongruent (Turnbull) myths. There was no source congruence difference in right-wing participants (not even pre-fact-check; *post hoc* contrast: *F*_1,343_ = 2.75, *p* = 0.10).

Thus, with regard to H1, it was found that corrections strongly reduced myth beliefs; regarding the three subordinate research questions, we found that (a) pre-fact-check, source congruence was a predictor of myth belief but only in left-wing participants; and (b) no source congruence effects occurred post-fact-check: myth belief was reduced to an equally low level across conditions, and corrections that could be seen as worldview-threatening (i.e. corrections of myths from an attitude-congruent source) were *not* less effective. Thus, (c) corrections were equally effective in left- and right-wing participants, but only left-wing participants showed a pre-correction bias towards believing myths from a favoured source more than myths from a non-favoured source. This resulted in left-wing participants reducing their belief in myths from an attitude-congruent source (Shorten) more than their belief in myths form an incongruent source (Turnbull), which is the *opposite* of what would be expected if the response to a correction were driven by motivated cognition.^4^

### 6.3. Fact belief

**Figure 2.**
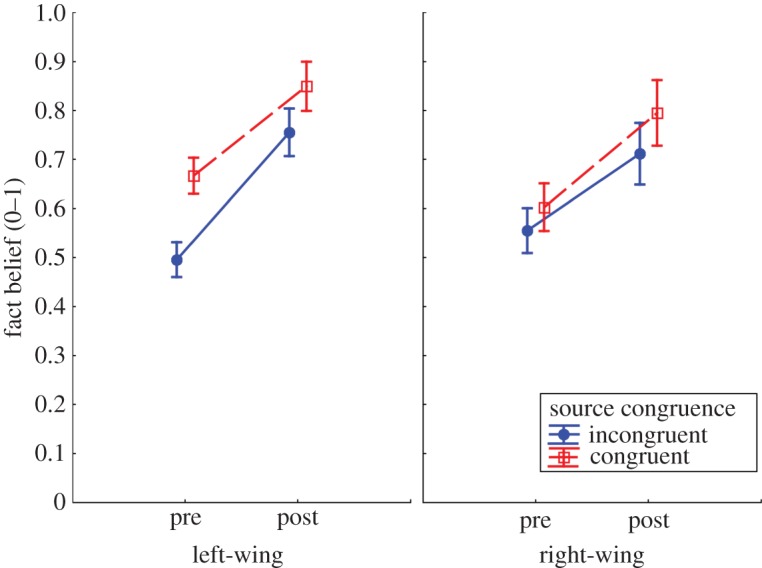
Pre- and post-fact-check fact belief across source congruence and political orientation conditions. Error bars indicate 95% confidence intervals.

As with myth belief, we ran an ANOVA on fact belief scores with the within-subjects factor fact-check (pre, post) and the between-subjects factors source congruence (congruent, incongruent) and political orientation (left-wing, right-wing). Table 1 shows the ANOVA results, and figure 2 shows the fact belief scores across conditions.

There was the expected main effect of fact-check, such that fact-checks increased fact belief, and a main effect of source congruence, such that facts were believed more if they came from an attitude-congruent source. There were no significant interactions, although a *post hoc* interaction contrast suggested that the congruence effect on pre-fact-check beliefs was again stronger in left-wing participants, *F*_1,343_ = 8.48, *p* = 0.004.

Thus, with regard to H1, it was found that fact-checks increased fact beliefs. With regard to the subordinate research questions, we found that (a) source–attitude congruence was a predictor of fact beliefs; and (b) fact-checks that could be seen as worldview-threatening (i.e. affirmations of myths from a non-favoured source) were *not* less effective, boosting fact belief by a similar amount across conditions (i.e. unlike the analysis of myth beliefs, there was still a congruence-based difference post-fact-check; *F*_1,343_ = 9.19, *p* = 0.003). Thus, by and large, (c) fact affirmations were equally effective in left- and right-wing participants.

### 6.4. Support

**Figure 3.**
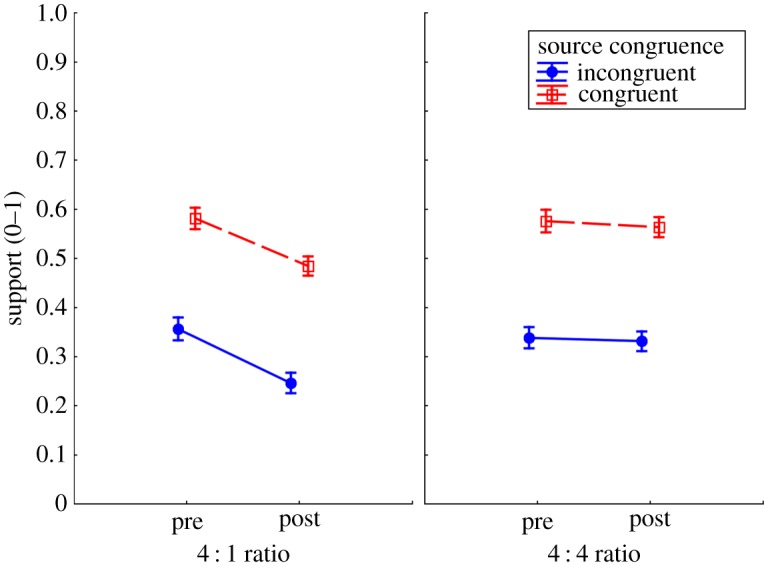
Pre- and post-fact-check support across source congruence and myth:fact ratio conditions. Error bars indicate 95% confidence intervals.

The composite support measure was analysed in a within–between ANOVA with the within-subjects factor fact-check (pre, post) and the between-subjects factors source congruence (congruent, incongruent) and myth:fact ratio (4 : 1, 4 : 4).^5^ Figure 3 shows the support scores across conditions.

Apart from the conceptually trivial main effect of source congruence, *F*_1,343_ = 149.52, *p* < 0.001, $\eta_{p}^{2}=0.85,$ indicating greater support for the favoured politician, the analysis yielded significant main effects of fact-check, *F*_1,343_ = 53.16, *p* < 0.001, $\eta_{p}^{2}=0.13,$ and myth:fact ratio, *F*_1,343_ = 4.18, *p* = 0.04, $\eta_{p}^{2}=0.01.$ These were qualified by a fact-check × myth:fact ratio interaction, *F*_1,343_ = 31.41, *p* < 0.001, $\eta_{p}^{2}=0.08,$ indicating that fact-checks reduced support—but only in the 4 : 1 condition (*F*_1,343_ = 83.87, *p* < 0.001) not the 4 : 4 condition (*F*_1,343_ = 1.41, *p* = 0.236). Thus, with regard to our second hypothesis (H2), it was found that candidate support did not change when an equal number of facts and myths were fact-checked (as in Swire *et al*. [39]), but support was reduced when participants received mostly myth corrections.

To better understand the interplay between perceived veracity, feelings and voting intentions, we ran a mediation analysis [60] to test the notion that at time 2 (post-manipulation), perceived veracity affected voting intentions through an indirect effect on feelings. We found that perceived veracity was a significant predictor of voting intentions, *t*_368_ = 17.41, *p* < 0.001 (*R*^2^ = 0.45). Perceived veracity also predicted feelings, *t*_368_ = 20.74, *p* < 0.001 (*R*^2^ = 0.54). When entering perceived veracity and feelings as concurrent predictors of voting intentions, feelings predicted voting intentions, *t*_367_ = 23.47, *p* < 0.001, but perceived veracity was no longer a significant predictor, *t*_367_ = 1.44, *p* = 0.15 (*R*^2^ = 0.78). A Sobel test showed that the drop in prediction when entering feelings as the mediator was significant, *Z* = 16.37, *p* < 0.001. This is consistent with an indirect effect of perceived veracity on voting intentions via feelings.

We also tested correlations between the shift in beliefs with the shift in support. Overall, the change in myth beliefs correlated significantly with the change in the composite support score, *r* = 0.346, *p* < 0.001. Overall change in fact beliefs did not correlate with the change in the composite support score, *r* = 0.075, *p* = 0.17. This outcome differs from the comparable immediate-test condition of the Swire *et al*. study, where the overall correlations between both myth belief or fact belief change and a composite score of feelings and voting intentions were small and non-significant. The pattern obtained in the present study was consistent across both political groups, left: *r* = 0.314, *p* < 0.001; right: *r* = 0.402, *p* < 0.001 for myth belief change; both *r* < 0.11, *p* > 0.21 for fact belief change. The correlation between myth belief change and support change was numerically larger in the 4 : 1 condition, *r* = 0.434, *p* < 0.001, than the 4 : 4 condition, *r* = 0.250, *p* = 0.001. A correlation between fact belief change and support change was present only in the 4 : 4 condition, *r* = 0.311, *p* < 0.001.

## 7. Discussion

The main result regarding myth belief and fact belief change was that participants updated their beliefs in accordance with the information received: affirmations increased fact beliefs, and refutations reduced myth beliefs. There was no evidence of motivated reasoning, and in fact, refutations reduced beliefs in myths *more strongly* if the myths came from a supported politician—that is, when there was congruence between the party affiliation of the participant and the politician (as discussed earlier, this counter-motivational effect was driven mainly by left-wing participants). The main result regarding politician support was that support decreased when mostly false statements were fact-checked (the 4 : 1 condition) but not when participants received an identical number of facts and myths (the 4 : 4 condition).

### 7.1. No attitude effects

The finding that pre-existing attitudes did not moderate belief change appears consistent with the attitude-protection hypothesis—that is, that motivated rejection of corrections occurs only for corrections which directly challenge strong attitudes [18,23]. While we used real statements from Shorten and Turnbull, we did not ensure that these statements reflected positions typically associated with each politician's party. Even if they happened to do so, participants may not have shared their preferred party's positions regarding the specific issues addressed (see [40–44]). Thus, it is unlikely that all of the fact-checks in the present study directly challenged strongly held convictions.

Future studies could contrast the impact of fact-checks relating to trivial or obscure topics with the impact of fact-checks relating to topics that are important and central to a politician's platform, while measuring participants' topic-specific attitudes. If attitude effects were found when strong beliefs are challenged, this would both support the attitude-protection hypothesis and reveal a limitation to fact-checking's effectiveness under such conditions.

### 7.2. Motivated reasoning not stronger among conservatives

There was no evidence that right-wing individuals showed stronger motivated reasoning or were any more likely to persist believing in inaccurate information. However, as noted, the fact-checks used in this study were unlikely to challenge strong beliefs. The processing of such fact-checks may be unaffected by variables—such as dogmatism, need for closure and sensitivity to threat—on which conservatives tend to score high. Thus, the argument that motivated reasoning is often stronger among right-wing people—due to their higher levels of those three traits [45–49]—would not necessarily apply under the present conditions [50].

While neither left- nor right-wing participants showed evidence of bias in changing their beliefs, the left-wing group appeared somewhat biased in their initial beliefs: initial belief was higher for statements from the preferred compared to the non-preferred politician. It should be noted in this context that the present study sacrificed some experimental control in order to use real statements from real politicians. Thus, it is possible that left-wing participants' apparent greater bias resulted from differences between the statements and politicians. For example, it may be that Turnbull is generally considered less credible than Shorten by partisans of both stripes, thus inflating the apparent bias of left-wing participants and deflating that of right-wing participants. Additionally, the two politicians held different positions—Turnbull was the prime minister at the time of testing, while Shorten was the leader of the opposition—which could have affected the results, for example, if the public generally applies higher standards to office holders and views them with greater scepticism than opposition leaders. Of course, statements differed, too, so the apparent asymmetry may have been caused by such idiosyncrasies.

### 7.3. Changes in support

The effects of fact-checking on support differed with the ratio of true and false statements. When there was an equal number of myths and facts, support was unaffected by fact-checking (cf. [39]). Moreover, balanced fact-checking had no impact on measures of support even when participants evaluated non-preferred politicians; these results can thus not be explained by motivated reasoning, which would only prevent a decline in attitudes towards preferred politicians [28]. Rather, a balanced sample of true and false statements may simply not substantially change the available evidence base or violate expectations. By contrast, when fact-checks involved primarily myths (four myths but only one fact), participants’ support did decline, despite the absence of attitude effects on belief change, and irrespective of whether the politician was from a participant's preferred party. It seems most likely that this change in support was caused by the cumulative impact of evidence that the politician made many incorrect statements, with perceived veracity affecting voting intentions via an impact on participants' feelings towards the respective politician. Thus, it appears that veracity does matter to voters, but that perceived veracity will be unaffected by a balance of positive and negative fact-checks.

These findings contrast with those of a follow-up study conducted in the USA by B Swire, A Berinsky, S Lewandowsky, UKH Ecker (2018, unpublished data) using statements from Donald Trump and Bernie Sanders. In that study, while balanced fact-checking of four true and four false statements again did not affect feelings, if mostly false statements from a politician were fact-checked, this caused a statistically significant decline in feelings towards that politician—in replication of the present results—although the magnitude of the overall effect was minute: Swire *et al*. observed an effect size of $\eta_{p}^{2}=0.02$ (an average decrease from 0.45 to 0.43 on a 0–1 scale), whereas the corresponding effect on feelings in the present study was $\eta_{p}^{2}=0.23$ (a decrease from 0.48 to 0.39). Thus, the main difference across studies was that Australians reduced their feelings towards politicians when discovering that 80% of their statements were untrue, whereas Americans’ feelings hardly shifted.

The reason for this discrepancy may relate to the fact that Australian politics is less polarized than US politics [61] and feelings for non-supported politicians in the US study were already relatively close to the floor (B Swire, A Berinsky, S Lewandowsky, UKH Ecker 2018, unpublished data). An interesting question in this context concerns the ‘magnitude’ of the political lies: the untruths disseminated by Bill Shorten and Malcolm Turnbull seem to pale in comparison to some of the disinformation spread by Donald Trump in particular [62]. Absent a quantification of the ‘magnitude’ of false statements, it is difficult to ascertain how this variable affected the results of the present study in comparison to the studies of Swire *et al*. [39] and B Swire, A Berinsky, S Lewandowsky, UKH Ecker (2018, unpublished data). However, we note that this factor makes the discrepancy between studies—namely that Australian voters reduced their feelings after the fact-checking of four relatively ‘small’ lies (and one fact), whereas many US voters did not substantially reduce feelings after the fact-checking of four more substantial lies (and one fact)—all the more remarkable.

Ultimately, the present results suggest that fact-checking could serve as a genuine threat to the electability of politicians who regularly make false statements. This threat could, in turn, decrease the frequency with which politicians spread misinformation. In support, Nyhan & Reifler [63] found evidence suggesting that US state representatives made fewer inaccurate statements if they were reminded of the presence of fact-checkers in their state, and of the potential electoral or reputational consequences of receiving a negative fact-check rating. That being said, the ‘threat potential’ of fact-checking may be greater in countries with compulsory voting (such as Australia) in comparison to countries with voluntary voting, where dissatisfaction from negative fact-checks may cause people to abstain from voting.

Moreover, it should be noted that this study does not establish how durable any changes in beliefs, voting intentions, feelings or perceived veracity might be, as these variables were measured immediately after the presentation of fact-checks. In reality, there will often be a longer delay between people encountering fact-checks and making decisions regarding policies or politicians, and the impact of fact-checking on beliefs is likely to at least partially fade over this time [6,39,64] (but see also [9]). If beliefs partially revert to their initial positions over time, it may be that attitudes would, too. However, it is also plausible that people could forget the details of the fact-checks—that is, what the fact-checks said about the topics addressed—while remembering that they mostly indicated the politician was incorrect. If so, the impact of fact-checks on attitudes could remain fairly stable. It would be valuable for future studies to provide more clarity about how the impact of fact-checks changes over time by measuring beliefs and attitudes after a delay. Finally, it is also unknown how much of a reduction in feelings and support is needed to actually change a vote; illuminating this relation could also be a target for future research.

## 8. Conclusion

Altogether, this study's findings are encouraging regarding both the potential effectiveness of fact-checking and the importance of veracity to voters. This is particularly so because we used real statements from real politicians, thus providing an externally valid test of whether fact-checks can change beliefs and supports, and whether this can occur unimpeded by initial attitudes [6,28,58]. That said, in this study, participants were unable to avoid fact-checks or to select which ones they received. In reality, some people may not encounter any fact-checks at all [9], and the sample of fact-checks which others encounter is often influenced by selective exposure and selective sharing [65,66]. Nevertheless, this study adds to the body of evidence (e.g. [9,36,37,63,67]) indicating that, to the extent that fact-checks are encountered, they have the potential to contribute to the functioning of democratic societies.

## Supplementary Material

Materials; supplementary table and figures in docx

## Supplementary Material

Data in csv format

## Footnotes

1 One could view the attitude-protection hypothesis as a special case of Kahan's identity-protection hypothesis (e.g. [27]), as attitudes are an integral part of a person's social identity. However, the emphasis here lies more on the premise that attitude-incongruent corrections may be fully effective as long as they do not directly call for attitude change.

2 Specifically, 175 participants were recruited via Qualtrics, 53 via Prolific Academic, 50 via Microworkers and 10 via Amazon Mechanical Turk.

3 This ANOVA, as well as the ANOVAs discussed below for the other dependent variables, was also run with participant source (laboratory, online) as an additional between-subjects factor. However, as no substantial differences in result patterns were found between the two sources, all analyses will be reported without that factor. All effects reported as significant in the following analyses involving political orientation were also significant in ANOVAs defining political orientation via median split (i.e. with approx. equal sample sizes across groups). Note that additional analyses including the ratio factor are reported in electronic supplementary material, table S1 and figure S1.

4 Despite the fact that groups were based on a midpoint-split, due to the overall skew of the sample towards the left, the left-wing group on average also held more extreme attitudes than the right-wing group. To explore whether our findings reflected a genuine left versus right asymmetry or simply stronger bias in the more extreme group, we added an extremity score as a covariate to the analysis (absolute distance to the scale midpoint, mean-centred) and found that the significant three-way interaction remained significant, suggesting a left- versus right-wing bias in pre-correction belief in myths from attitude-congruent versus incongruent sources.

5 An analysis including the political orientation factor can be found in electronic supplementary material, table S1. Moreover, for the interested reader, the results of separate ANOVAs on the three component measures can also be found in the electronic supplementary material, table S2; mean voting intentions, feelings and perceived veracity scores across conditions can be found in electronic supplementary material, figures S2–S4, respectively. Overall, the three measures behaved very similarly in response to the experimental manipulations.

## Data accessibility

The data and experimental materials are provided as electronic supplementary material.

## References

[1] Birch S, Allen N 2010 How honest do politicians need to be? Polit. Q. 81, 49– 56. (10.1111/j.1467-923X.2010.02066.x)

[2] Duelfer C 2004 Comprehensive report of the special advisor to the DCI on Iraq's WMD. Langley, VA: Central Intelligence Agency.

[3] Duelfer C 2016 WMD elimination in Iraq, 2003. Nonprolif. Rev. 23, 163–184. (10.1080/10736700.2016.1179431)

[4] Gaines BJ, Kuklinski JH, Quirk PJ, Peyton B, Verkuilen J 2007 Same facts, different interpretations: partisan motivation and opinion on Iraq. J. Politics 69, 957–974. (10.1111/j.1468-2508.2007.00601.x)

[5] Nyhan B, Reifler J 2010 When corrections fail: the persistence of political misperceptions. Polit. Behav. 32, 303–330. (10.1007/s11109-010-9112-2)

[6] Berinsky AJ 2017 Rumors and health care reform: experiments in political misinformation. Br. J. Polit. Sci. 47, 241–262. (10.1017/S0007123415000186)

[7] Nyhan B, Reifler J 2012 *Misinformation and fact-checking: research findings from social science: prepared for New America Foundation*. See http://www.dartmouth.edu/~nyhan/Misinformation_and_Fact-checking.pdf

[8] Marietta M, Barker DC, Bowser T 2015 Fact-checking polarized politics: does the fact-check industry provide consistent guidance on disputed realities? Forum 13, 577–596. (10.1515/for-2015-0040)

[9] Nyhan B, Reifler J 2015 Estimating fact-checking's effects: evidence from a long term experiment during campaign 2014. Washington, DC: American Press Institute.

[10] Ajzen I 2001 Nature and operation of attitudes. Annu. Rev. Psychol. 52, 27–58. (10.1146/annurev.psych.52.1.27) 11148298

[11] Hogg MA, Smith JR 2007 Attitudes in social context: a social identity perspective. Eur. Rev. Soc. Psychol. 18, 89–131. (10.1080/10463280701592070)

[12] Ecker UK, Hogan JL, Lewandowsky S 2017 Reminders and repetition of misinformation: helping or hindering its retraction? J. Appl. Res. Mem. Cogn. 6, 185–192. (10.1016/j.jarmac.2017.01.014)

[13] Ecker UK, Lewandowsky S, Apai J 2011 Terrorists brought down the plane!—No, actually it was a technical fault: processing corrections of emotive information. Q. J. Exp. Psychol. 64, 283–310. 10.1080/17470218.2010.497927 20694936

[14] Guillory JJ, Geraci L 2010 The persistence of inferences in memory for younger and older adults: remembering facts and believing inferences. Psychon. Bull. Rev. 17, 73–81. (10.3758/PBR.17.1.73) 20081164

[15] Guillory JJ, Geraci L 2013 Correcting erroneous inferences in memory: the role of source credibility. J. Appl. Res. Mem. Cogn. 2, 201–209. (10.1016/j.jarmac.2013.10.001)

[16] Johnson HM, Seifert CM 1994 Sources of the continued influence effect: when misinformation in memory affects later inferences. J. Exp. Psychol. Learn. Mem. Cogn. 20, 1420–1436.

[17] Johnson HM, Seifert CM 1998 Updating accounts following a correction of misinformation. J. Exp. Psychol. Learn. Mem. Cogn. 24, 1483–1494. 9835062 10.1037//0278-7393.24.6.1483

[18] Ecker UK, Lewandowsky S, Fenton O, Martin K 2014 Do people keep believing because they want to? Preexisting attitudes and the continued influence of misinformation. Mem. Cognit. 42, 292–304. 10.3758/s13421-013-0358-x 24005789

[19] Lewandowsky S, Ecker UK, Seifert CM, Schwarz N, Cook J 2012 Misinformation and its correction: continued influence and successful debiasing. Psychol. Sci. Public Interest 13, 106–131. (10.1177/1529100612451018) 26173286

[20] Schwarz N, Newman E, Leach W 2016 Making the truth stick & the myths fade: lessons from cognitive psychology. Behav. Sci. Pol. 2, 85–95. (10.1353/bsp.2016.0009)

[21] Seifert CM 2002 The continued influence of misinformation in memory: what makes a correction effective? Psychol. Learn. Motiv. 41, 265–292. (10.1016/S0079-7421(02)80009-3)

[22] Cook J, Lewandowsky S 2016 Rational irrationality: modeling climate change belief polarization using Bayesian networks. Top. Cogn. Sci. 8, 160–179. (10.1111/tops.12186) 26749179

[23] Ecker UK, Ang LC In press. Political attitudes and the processing of misinformation corrections. Polit. Psychol. (10.1111/pops.12494)

[24] Hart PS, Nisbet EC 2012 Boomerang effects in science communication: how motivated reasoning and identity cues amplify opinion polarization about climate mitigation policies. Commun. Res. 39, 701–723. (10.1177/0093650211416646)

[25] Nyhan B, Reifler J, Ubel PA 2013 The hazards of correcting myths about health care reform. Med. Care 51, 127–132. (10.1097/MLR.0b013e318279486b) 23211778

[26] Redlawsk DP 2002 Hot cognition or cool consideration? Testing the effects of motivated reasoning on political decision making. J. Polit. 64, 1021–1044. (10.1111/1468-2508.00161)

[27] Kahan DM 2013 Ideology, motivated reasoning, and cognitive reflection. Judgm. Decis. Mak. 8, 407–424.

[28] Redlawsk DP, Civettini AJ, Emmerson KM 2010 The affective tipping point: do motivated reasoners ever ‘get it’? Polit. Psychol. 31, 563–593. (10.1111/j.1467-9221.2010.00772.x)

[29] Meffert MF, Chung S, Joiner AJ, Waks L, Garst J 2006 The effects of negativity and motivated information processing during a political campaign. J. Commun. 56, 27–51.

[30] Nisbet EC, Cooper KE, Garrett RK 2015 The partisan brain: how dissonant science messages lead conservatives and liberals to (dis)trust science. Ann. Am. Acad. Polit. Soc. Sci. 658, 36–66. (10.1177/0002716214555474)

[31] Prasad M, Perrin AJ, Bezila K, Hoffman SG, Kindleberger K, Manturuk K, Powers AS 2009 ‘There must be a reason’: Osama, Saddam, and inferred justification. Sociol. Inq. 79, 142–162. (10.1111/j.1475-682X.2009.00280.x)

[32] Taber CS, Lodge M 2006 Motivated skepticism in the evaluation of political beliefs. Am. J. Polit. Sci. 50, 755–769. (10.1111/j.1540-5907.2006.00214.x)

[33] Garrett RK, Weeks BE 2013 The promise and peril of real-time corrections to political misperceptions. In Proc. 2013 Conf. Computer Supported Cooperative Work, pp. 1047–1058. New York, NY: ACM (10.1145/2441776.2441895)

[34] Garrett RK, Nisbet EC, Lynch EK 2013 Undermining the corrective effects of media-based political fact checking? The role of contextual cues and naïve theory. J. Commun. 63, 617–637.

[35] Pomerantz EM, Chaiken S, Tordesillas RS 1995 Attitude strength and resistance processes. J. Pers. Soc. Psychol. 69, 408–419. 7562388 10.1037//0022-3514.69.3.408

[36] Weeks BE 2015 Emotions, partisanship, and misperceptions: how anger and anxiety moderate the effect of partisan bias on susceptibility to political misinformation. J. Commun. 65, 699–719.

[37] Weeks BE, Garrett RK 2014 Electoral consequences of political rumors: motivated reasoning, candidate rumors, and vote choice during the 2008 US presidential election. Int. J. Public Opin. Res. 26, 401–422.

[38] Zuwerink JR, Devine PG 1996 Attitude importance and resistance to persuasion: it's not just the thought that counts. J. Pers. Soc. Psychol. 70, 931–944.

[39] Swire B, Berinsky AJ, Lewandowsky S, Ecker UKH 2017 Processing political misinformation: comprehending the Trump phenomenon. R. Soc. open sci. 4, 160802 (10.1098/rsos.160802) 28405366 PMC5383823

[40] Carsey TM, Layman GC 2006 Changing sides or changing minds? Party identification and policy preferences in the American electorate. Am. J. Polit. Sci. 50, 464–477. (10.1111/j.1540-5907.2006.00196.x)

[41] Feldman S, Johnston C 2014 Understanding the determinants of political ideology: implications of structural complexity. Polit. Psychol. 35, 337–358. (10.1111/pops.12055)

[42] Goren P 2005 Party identification and core political values. Am. J. Polit. Sci. 49, 881–896. (10.1111/j.1540-5907.2005.00161.x)

[43] MacCoun RJ, Paletz S 2009 Citizens' perceptions of ideological bias in research on public policy controversies. Polit. Psychol. 30, 43–65. (10.1111/j.1467-9221.2008.00680.x)

[44] Weeden J, Kurzban R 2016 Do people naturally cluster into liberals and conservatives? Evol. Psychol. Sci. 2, 47–57. (10.1007/s40806-015-0036-2)

[45] Hibbing JR, Smith KB, Alford JR 2014 Differences in negativity bias underlie variations in political ideology. Behav. Brain Sci. 37, 297–350. (10.1017/S0140525X13001192) 24970428

[46] Jost JT, Glaser J, Kruglanski AW, Sulloway FJ 2003 Political conservatism as motivated social cognition. PsyB 129, 339–375. 10.1037/0033-2909.129.3.339 12784934

[47] Jost JT, Napier JL, Thorisdottir H, Gosling SD, Palfai TP, Ostafin B 2007 Are needs to manage uncertainty and threat associated with political conservatism or ideological extremity? Pers. Soc. Psychol. Bull. 33, 989–1007. (10.1177/0146167207301028) 17620621

[48] Jost JT 2017 Ideological asymmetries and the essence of political psychology. Polit. Psychol. 38, 167–208. (10.1111/pops.12407)

[49] Onraet E, Van Hiel A, Roets A, Cornelis I 2011 The closed mind: ‘experience’ and ‘cognition’ aspects of openness to experience and need for closure as psychological bases for right-wing attitudes. Eur. J. Pers. 25, 184–197. (10.1002/per.775)

[50] Nam HH, Jost JT, Van Bavel JJ 2013 ‘Not for all the tea in China!’ Political ideology and the avoidance of dissonance-arousing situations. PLoS ONE 8, e59837 (10.1371/journal.pone.0059837) 23620724 PMC3631191

[51] Pennycook G, Cheyne JA, Barr N, Koehler DJ, Fugelsang JA 2015 On the reception and detection of pseudo-profound bullshit. Judgm. Decis. Mak. 10, 549–563.

[52] Pfattheicher S, Schindler S 2016 Misperceiving bullshit as profound is associated with favorable views of Cruz, Rubio, Trump and conservatism. PLoS ONE 11, e0153419 (10.1371/journal.pone.0153419) 27128318 PMC4851308

[53] Sterling J, Jost JT, Pennycook G 2016 Are neoliberals more susceptible to bullshit? Judgm. Decis. Mak. 11, 352–360.

[54] Ditto PH, Liu B, Clark CJ, Wojcik SP, Chen EE, Grady RH, Zinger JF 2018 At least bias is bipartisan: a meta-analytic comparison of partisan bias in liberals and conservatives. Perspect. Psychol. Sci. 7, 496–503. (10.1177/1745691617746796) 29851554

[55] Lewandowsky S, Oberauer K 2016 Motivated rejection of science. Curr. Dir. Psychol. Sci. 25, 217–222. (10.1177/0963721416654436)

[56] Kahan DM, Jenkins-Smith H, Braman D 2011 Cultural cognition of scientific consensus. J. Risk Res. 14, 147–174. (10.1080/13669877.2010.511246)

[57] Washburn AN, Skitka LJ In press. Science denial across the political divide. Soc. Psychol. Personal Sci. (10.1177/1948550617731500)

[58] Thorson E 2016 Belief echoes: the persistent effects of corrected misinformation. Polit. Commun. 33, 460–480. (10.1080/10584609.2015.1102187)

[59] Mehrabian A 1996 Relations among political attitudes, personality, and psychopathology assessed with new measures of libertarianism and conservatism. Basic Appl. Soc. Psych. 18, 469–491. (10.1207/s15324834basp1804_7)

[60] Baron RM, Kenny DA 1986 The moderator-mediator variable distinction in social psychological research: conceptual, strategic, and statistical considerations. J. Pers. Soc. Psychol. 51, 1173–1182. 3806354 10.1037//0022-3514.51.6.1173

[61] Reilly B 2016 Democratic design and democratic reform: the case of Australia. Taiwan J. Democracy 12, 1–16.

[62] Lewandowsky S, Ecker UK. H., Cook J 2017 Beyond misinformation: understanding and coping with the post-truth era. J. Appl. Res. Mem. Cogn. 6, 353–369. (10.1016/j.jarmac.2017.07.008)

[63] Nyhan B, Reifler J 2015 The effect of fact-checking on elites: a field experiment on US state legislators. Am. J. Polit. Sci. 59, 628–640. (10.1111/ajps.12162)

[64] Swire B, Ecker UKH, Lewandowsky S 2017 The role of familiarity in correcting inaccurate information. J. Exp. Psychol. Learn. Mem. Cogn. 43, 1948–1961. (10.1037/xlm0000422) 28504531

[65] Shin J, Thorson K 2017 Partisan selective sharing: the biased diffusion of fact-checking messages on social media. J. Commun. 67, 233–255. (10.1111/jcom.12284)

[66] Frimer JA, Skitka LJ, Motyl M 2017 Liberals and conservatives are similarly motivated to avoid exposure to one another's opinions. J. Exp. Soc. Psychol. 72, 1–12. (10.1016/j.jesp.2017.04.003)

[67] Wood T, Porter E In press. The elusive backfire effect: mass attitudes’ steadfast factual adherence. Polit. Behav. (10.1007/s11109-018-9443-y)

