# Supplementary material for: Does truth matter to voters? The effects of correcting political misinformation in an Australian sample
Source: R Soc Open Sci. 2018 Dec 19;5(12):180593. doi: 10.1098/rsos.180593 (PMC6304148; doi:10.1098/rsos.180593)
Supplement: Materials; supplementary table and figures in docx [file rsos180593supp1.docx]

**Supplement**

**Myths, Facts, and Fact-Checks Used**

| *Shorten’s Myths and the Associated Fact-Checks* | | |
| --- | --- | --- |
| Item Number | Item | Fact-Check |
| Shorten myth 1 | Bill Shorten said nine out of ten Australians spend more than 90 minutes a day travelling to and from work. | Shorten’s statement is incorrect and misleading. Data from the ‘Household, Income and Labour Dynamics in Australia’ survey show that less than two in ten Australians spend more than 90 minutes a day travelling to and from work. The average time spent commuting was around half that time. |
| Shorten myth 2 | Bill Shorten said in 2015 that the Liberals had cut $1 billion from childcare. | Shorten’s statement is incorrect and misleading. Budget papers show the Liberals increased overall funding to many of the programs Shorten suggested were cut, as well as funding new childcare programs. Moreover, some programs Shorten counted as being cut were simply due to expire anyway under agreements from the previous Labor government. Thus, there was no $1 billion cut to childcare spending by the Liberals. |
| Shorten myth 3 | Bill Shorten said that scrapping the GST on tampons would require the agreement of state premiers. | Shorten’s statement is incorrect and misleading. The Australian Constitution grants the power to make laws regarding taxation to the federal government, not to state governments. Therefore the federal government can change or remove the GST on tampons by itself. Experts say it would be unconstitutional to require the federal government to get the agreement of state premiers for this. |
| Shorten myth 4 | Bill Shorten said the Liberals’ 2016-2017 federal budget would give people earning over a million dollars per year a $17,000 tax cut. | Shorten’s statement is incorrect and misleading. The 2016-2017 budget contains only a negligible tax cut (about $300) for people earning over a million. The tax reduction for these people results almost entirely from the expiry of a temporary budget-repair levy brought in by the Liberal government in 2014. Thus, the tax rates will essentially return to the pre-2014 baseline, and this has nothing to do with the 2016-2017 budget. |

| *Shorten’s Facts and the Associated Fact-Checks* | | |
| --- | --- | --- |
| Item Number | Item | Fact-Check |
| Shorten fact 1 | Bill Shorten said in 2016 that over one million Australians are underemployed. | Shorten’s statement is correct. People are considered underemployed when they work at least one hour per week, but would like to be working more and are capable of doing so. The Australian Bureau of Statistics estimated that the number of underemployed people in 2016 was 1,063,800. |
| Shorten fact 2 | Bill Shorten said 2.5 million Australians live below the poverty line. | Shorten’s statement is correct. A report from the Australian Council of Social Service estimated that approximately 2.55 million Australians live below the poverty line. Additionally, research from the National Centre for Social and Economic Modelling provides a very similar estimate, suggesting 2.6 million Australians live below the poverty line. |
| Shorten fact 3 | Bill Shorten said in 2016 that $100 billion had been added to Australia’s national debt under the Liberal government. | Shorten’s statement is correct. The ‘Pre-Election Economic and Fiscal Outlook’ papers for 2013 and 2016 show that, between the Coalition winning government and Shorten making this statement, Australia’s net debt increased from $184 billion to $285.8 billion. The 2016-2017 ‘Budget Paper 1’ also supports the claim of such a rise in net debt levels. |
| Shorten fact 4 | Bill Shorten said in late 2014 that the youth unemployment rate was at a 13-year high. | Shorten’s statement is correct. The Australian Bureau of Statistics report that in October 2014, the youth unemployment rate was at 13.8%. The last time it had been as high as 13.8% was in November 2001, 13 years prior to Shorten’s statement. |

| *Turnbull’s Myths and the Associated Fact-Checks* | | |
| --- | --- | --- |
| Item Number | Item | Fact-Check |
| Turnbull myth 1 | Malcolm Turnbull said that Australia’s target for reduction of per capita greenhouse gas emissions is second only to Brazil’s. | Turnbull’s statement is incorrect and misleading. Multiple countries in addition to Brazil have higher emission reduction targets than Australia, including Switzerland and Norway. In addition, experts highlight that Australia’s emissions reductions targets must be considered in the context of Australia having one of the highest per capita emission rates to start with, while many other countries have already reduced their emissions. |
| Turnbull myth 2 | Malcolm Turnbull suggested the 2016 blackout in South Australia was related to the state’s use of renewable energy, stating that the blackout should serve as a wake-up call regarding unrealistic renewable energy targets. | Turnbull’s statement is incorrect and misleading. The blackout had nothing to do with the proportion of renewable energy in South Australia’s electricity supply. It actually resulted from a severe storm in South Australia causing transmission lines to fall over. Emails released under freedom-of-information rules show Turnbull’s officials had been advised of this the day after the blackout by the Australian Energy Market Operator. |
| Turnbull myth 3 | In arguing that the Australian Building and Construction Commission (ABCC) needed to re-established, Malcolm Turnbull stated that two thirds of all industrial disputes were in the construction sector.​ | Turnbull’s statement is incorrect and misleading. The claim “cherry-picks” an outlier—a single 2015 quarter in which the proportion of industrial disputes that were in the construction sector was much higher than usual. In truth, only about 30% of industrial disputes have been in the construction sector since the ABCC was abolished in March 2012. |
| Turnbull myth 4 | Malcolm Turnbull said the abolition of negative gearing in the 1980s caused rents to rise. | Turnbull’s statement is incorrect and misleading. Although negative gearing was abolished nation-wide, the only major cities where rents rose were Perth and Sydney, while those in Adelaide and Brisbane fell substantially. Experts argue that the increases in Perth and Sydney were caused by extremely low rental vacancies at the time, in addition to high interest rates. Also, rents in Sydney actually increased much more after negative gearing was reinstated. |
| *Turnbull’s Facts and the Associated Fact-Checks* | | |
| Item Number | Item | Fact-Check |
| Turnbull fact 1 | Malcolm Turnbull said that Australian farmers are among the least subsidised in the world. | Turnbull’s statement is correct. A report published by the Organisation for Economic Co-operation and Development (OECD) showed that Australian farmers were the second least subsidised in OECD countries, after only New Zealand, and that they were among the least subsidised in the world. |
| Turnbull fact 2 | Malcolm Turnbull said that the Labor party previously advocated cutting the tax rate for companies. | Turnbull’s statement is correct. Labor cut the company tax rate when it was in government between 1983 and 1996, and the Gillard Labor government promised in 2010 to do the same if re-elected. Senior members of the Gillard government advocated cutting the company tax rate on grounds that it would increase investment and job growth. |
| Turnbull fact 3 | Malcolm Turnbull said that around 300,000 jobs were created in Australia in 2015. | Turnbull’s statement is correct. The Australian Bureau of Statistics (ABS) reports that the number of employed people rose in 2015 by 301,300 by seasonally adjusted estimates. ABS trend estimates, which many experts regard as the better measure, suggest an even greater number of 312,100 jobs created in 2015. |
| Turnbull fact 4 | Malcolm Turnbull said in 2016 that globally there were more than 65 million people forcibly displaced. | Turnbull’s statement is correct. The United Nations Refugee Agency’s Global Trends report stated there were a total of 65.3 million people displaced at the end of 2015. This is based on data from governments, partner agencies, and the Refugee Agency itself. |

**Questions Presented to Participants**

*What is your age?*

*What is your gender?* (Answered with “Male”, “Female”, or “Other / Prefer not to say”.)

*I am politically more in line with the Labor party than the Liberal party.* (5-point Likert scale ranging from “Strongly disagree” to “Strongly agree”. Negatively-keyed. This and the following four items comprise Ecker and Ang’s [2017] party-preference scale.)

*In any election, given a choice between a Liberal and a Labor candidate, I will select the Liberal over the Labor candidate.* (5-point Likert scale ranging from “Strongly disagree” to “Strongly agree”.)

*I cannot see myself ever voting to elect Liberal candidates.* (5-point Likert scale ranging from “Strongly disagree” to “Strongly agree”. Negatively-keyed.)

*The major national media are too protective of the Labor party for my taste.* (5-point Likert scale ranging from “Strongly disagree” to “Strongly agree”.)

*On balance, I lean politically more to the left (Labor) than to the right (Liberal).* (5-point Likert scale ranging from “Strongly disagree” to “Strongly agree”. Negatively-keyed.)

*Please indicate the extent to which you identify as politically left-wing or right-wing.* (7-point Likert scale ranging from “Very left-wing” to “Very right-wing”.)

*Who is the leader of the political party One Nation?* (Multiple choice, with the options “Julia Gillard”, “Tony Abbott”, “Tanya Plibersek”, “Robert Menzies”, “Pauline Hanson”, “Kristina Keneally”, “Malcolm Fraser”, and “Peter Dutton”.)

*Which of these people is a former Australian Prime Minister?* (Multiple choice, with the options “Barnaby Joyce”, “Kevin Rudd”, “Anthony Albanese”, “Gareth Evans”, “Corey Bernardi”, “Edith Cowan”, “Julie Bishop”, and “Peter Garrett”.)

*If today was election day, and you could vote directly for a prime minister, how likely would you be to vote for Bill Shorten / Malcolm Turnbull?* (11-point Likert scale ranging from “Extremely unlikely” to “Extremely likely”. For this and the following questions, which name a given participant was shown was determined by which politician condition that participant had been allocated to.)

*We’d like to get your feelings toward Bill Shorten / Malcolm Turnbull using something we call the feeling thermometer. Ratings between 50 and 100 degrees mean that you feel favorably and warm toward Bill Shorten / Malcolm Turnbull; ratings between 0 and 50 degrees mean that you don’t feel favorably toward him and that you don't care too much for him. You would rate him at the 50 degree mark if you don’t feel particularly warm or cold toward him.* (Slider from 0 to 100.)

*On the whole, how often would you say Bill Shorten / Malcolm Turnbull is accurate in what he says?* (11-point Likert scale ranging from “Never” to “Always”.)

*On a scale between 0-10, do you believe Shorten’s statement to be true?* (11-point Likert scale ranging from “Definitely false” to “Definitely true”. Asked alongside each statement a participant is shown, and then again as each statement is repeated following the fact-checks.)

*If today was election day, and you could vote directly for a prime minister, how likely would you be to vote for Bill Shorten / Malcolm Turnbull?* (11-point Likert scale ranging from “Extremely unlikely” to “Extremely likely”. Repeat of prior question.)

*We’d again like to get your feelings toward Bill Shorten / Malcolm Turnbull using the feeling thermometer. Remember, ratings between 50 and 100 degrees mean that you feel favorably and warm toward Bill Shorten / Malcolm Turnbull; ratings between 0 and 50 degrees mean that you don’t feel favorably toward him and that you don’t care too much for him. You would rate him at the 50 degree mark if you don’t feel particularly warm or cold toward him.* (Slider from 0 to 100. Modification of prior question.)

*On the whole, how often would you say Bill Shorten / Malcolm Turnbull is accurate in what he says?* (11-point Likert scale ranging from “Never” to “Always”. Repeat of prior question.)

*What is the highest level of education you have completed?* (Multiple choice, with the options “Did not graduate from high school”, “High school graduate”, “TAFE or other non-university qualification”, “Some university, but no degree”, “3-year university degree”, “4-year university degree”, and “Postgraduate degree”.)

*It is important that we know whether you have actually taken the time to read the questions. Please select Shy to show you have been paying attention, and ignore the other response options.* (Multiple choice, with the options “Confused”, “Upset”, “Comfortable”, “Happy”, “Bored”, “Angry”, “Satisfied”, “Shy”, “Nervous”, “Threatened”, “Curious”, and “None of the above”.)

*Have you previously completed a similar version of this survey?* (Answered “Yes” or “No”.)

*As previously noted, the results of this experiment may have implications for psychological theory. Therefore, please truthfully answer the following question: In your honest opinion, should we use your data? This is not related to how well you think you performed, but whether you put in a* ***reasonable effort****. We guarantee that your response to this question will have* ***no effect on your SONA credit or your grade / your reimbursement or the approval of your assignment / your reimbursement for completing this survey****. We just want to know what data to include in our analyses. Thanks!* (Multiple choice, with the options “Yes; I put in a reasonable effort”, “Maybe; I was somewhat distracted”, and “No; I didn’t really pay any attention”. Participants recruited at the University of Western Australia saw “your SONA credit or your grade”; those recruited via Prolific Academic, Microworkers, or Mechanical Turk saw “your reimbursement or the approval of your assignment”; and those recruited via Qualtrics saw “your reimbursement for completing this survey”.)

|  | **Myth belief** | | |  | **Fact belief** | | |  | **Support** | | |
| --- | --- | --- | --- | --- | --- | --- | --- | --- | --- | --- | --- |
| **Effects** | ***F*(1,339)** | ***p*** | **η_p_^2^** |  | ***F*(1,339)** | ***p*** | **η_p_^2^** |  | ***F*(1,339)** | ***p*** | **η_p_^2^** |
| SC | 14.26 | <.001 | .04 |  | 25.54 | <.001 | .07 |  | 131.20 | <.001 | .28 |
| R | < 1 |  |  |  | 2.38 | .124 | .01 |  | 4.27 | .040 | .01 |
| PO | 5.24 | .023 | .02 |  | 1.41 | .236 | <.01 |  | < 1 |  |  |
| SC × R | 1.75 | .187 | <.01 |  | < 1 |  |  |  | < 1 |  |  |
| SC × PO | 3.20 | .075 | .01 |  | 2.21 | .138 | .01 |  | < 1 |  |  |
| R × PO | < 1 |  |  |  | 2.84 | .093 | .01 |  | < 1 |  |  |
| SC × R × PO | 2.33 | .128 | .01 |  | < 1 |  |  |  | < 1 |  |  |
| FC | 588.09 | <.001 | .63 |  | 159.99 | <.001 | .32 |  | 43.77 | <.001 | .11 |
| FC × SC | 17.72 | <.001 | .05 |  | < 1 |  |  |  | < 1 |  |  |
| FC × R | < 1 |  |  |  | < 1 |  |  |  | 25.93 | <.001 | .07 |
| FC × PO | < 1 |  |  |  | 1.69 | .195 | <.01 |  | 2.64 | .105 | .01 |
| FC × SC × R | 7.39 | .007 | .02 |  | < 1 |  |  |  | < 1 |  |  |
| FC × SC × PO | 8.33 | .004 | .02 |  | 3.81 | .052 | .01 |  | < 1 |  |  |
| FC × R × PO | 4.11 | .043 | .01 |  | 1.74 | .188 | .01 |  | 2.32 | .129 | .01 |
| FC × SC × R × PO | < 1 |  |  |  | 2.14 | .145 | .01 |  | < 1 |  |  |

Table S1.

*Note.* SC, source congruence; R, myth:fact ratio; PO, political orientation; FC, fact-check.

Table S2.

|  | **Voting intention** | | |  | **Feelings** | | |  | **Perceived veracity** | | |
| --- | --- | --- | --- | --- | --- | --- | --- | --- | --- | --- | --- |
| **Effects** | ***F*(1,339)** | ***p*** | **η_p_^2^** |  | ***F*(1,339)** | ***p*** | **η_p_^2^** |  | ***F*(1,339)** | ***p*** | **η_p_^2^** |
| SC | 147.29 | <.001 | .30 |  | 106.12 | <.001 | .24 |  | 82.59 | <.001 | .20 |
| R | 3.67 | .056 | .01 |  | 1.39 | .239 | <.01 |  | 8.03 | .005 | .02 |
| PO | < 1 |  |  |  | < 1 |  |  |  | < 1 |  |  |
| SC × R | < 1 |  |  |  | < 1 |  |  |  | < 1 |  |  |
| SC × PO | < 1 |  |  |  | 1.06 | .304 | <.01 |  | < 1 |  |  |
| R × PO | < 1 |  |  |  | < 1 |  |  |  | < 1 |  |  |
| SC × R × PO | 1.20 | .274 | <.01 |  | < 1 |  |  |  | < 1 |  |  |
| FC | 23.79 | <.001 | .07 |  | 40.71 | <.001 | .11 |  | 29.45 | <.001 | .08 |
| FC × SC | < 1 |  |  |  | 2.27 | .133 | .01 |  | < 1 |  |  |
| FC × R | 8.84 | .003 | .03 |  | 15.38 | <.001 | .04 |  | 32.79 | <.001 | .09 |
| FC × PO | < 1 |  |  |  | < 1 |  |  |  | 4.44 | .036 | .01 |
| FC × SC × R | 1.60 | .206 | <.01 |  | < 1 |  |  |  | < 1 |  |  |
| FC × SC × PO | < 1 |  |  |  | 2.15 | .143 | .01 |  | < 1 |  |  |
| FC × R × PO | 1.77 | .184 | .01 |  | 1.27 | .260 | <.01 |  | 1.68 | .196 | <.01 |
| FC × SC × R × PO | < 1 |  |  |  | 1.26 | .263 | <.01 |  | < 1 |  |  |

*Note.* SC, source congruence; R, myth:fact ratio; PO, political orientation; FC, fact-check.

Figure S1.


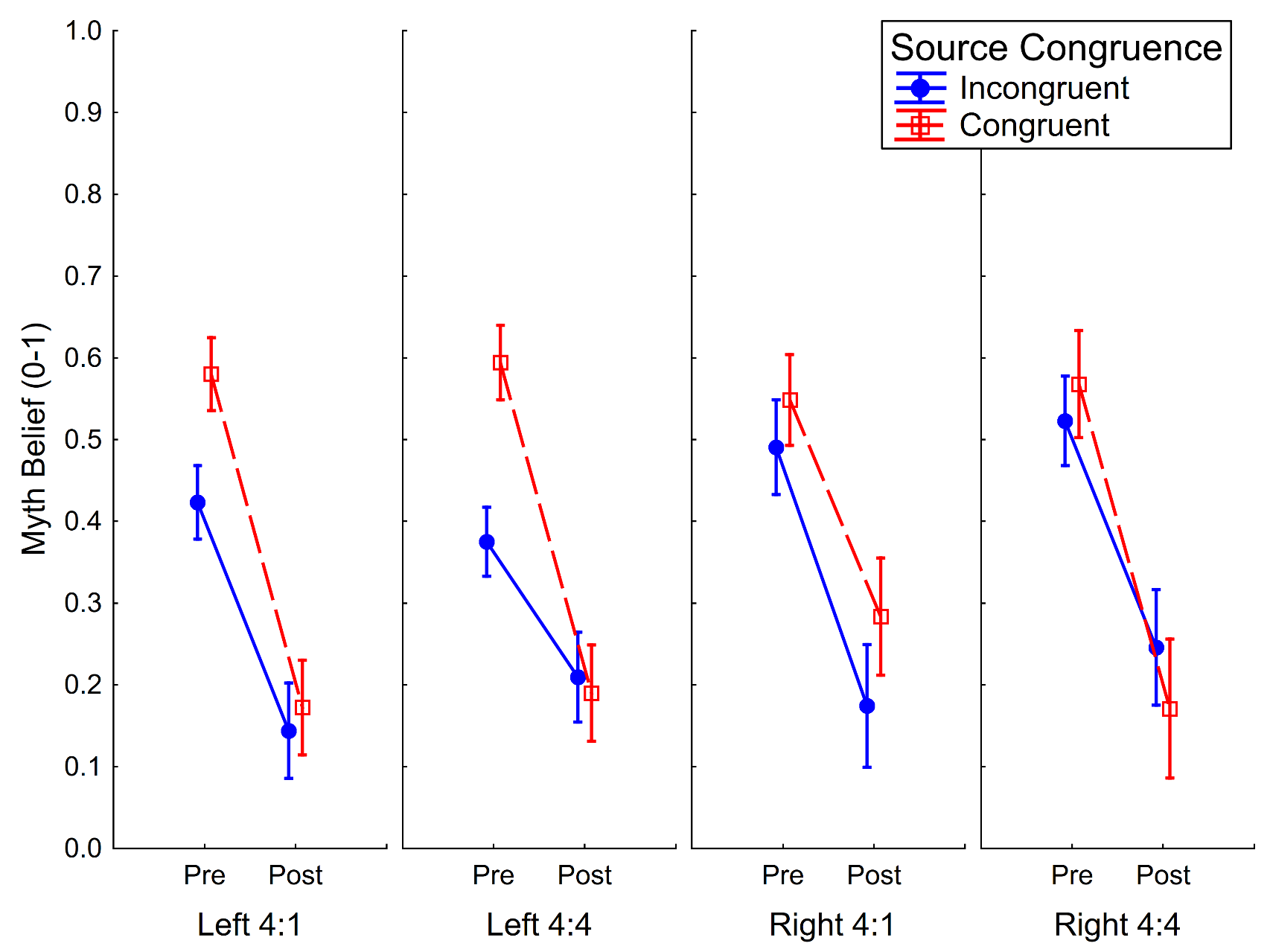


Figure S2.

**
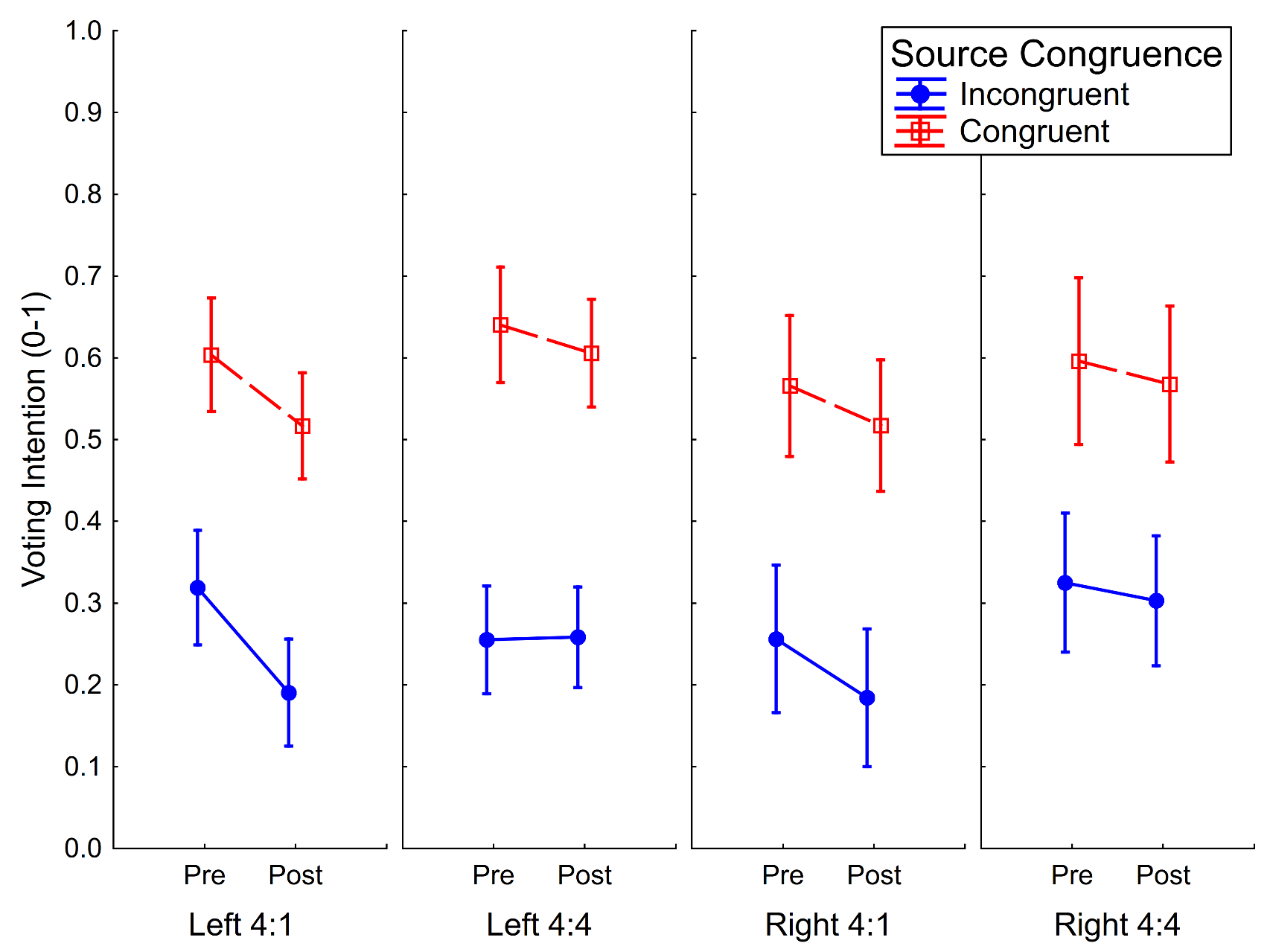
**

Figure S3.


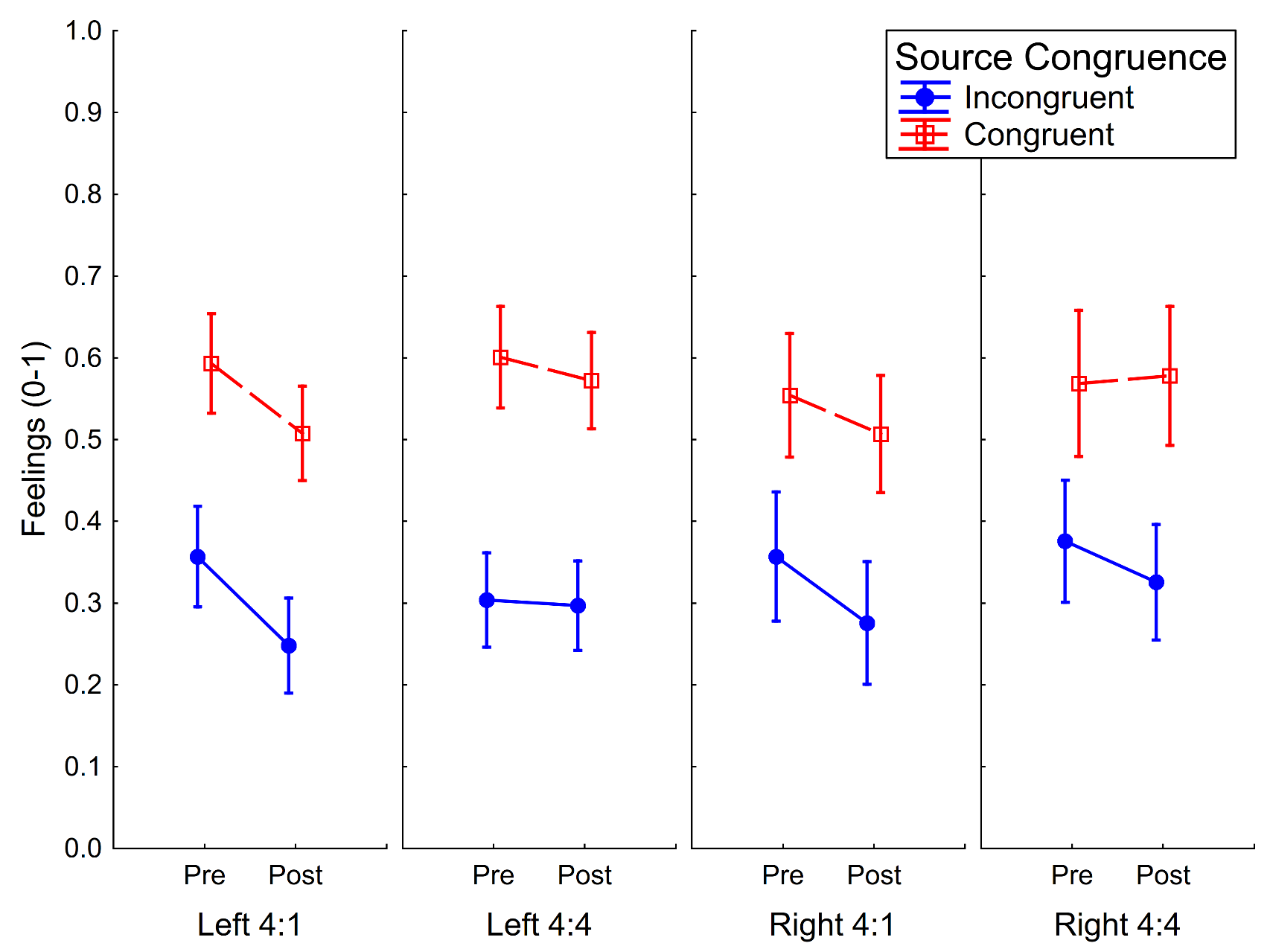


Figure S4.

**
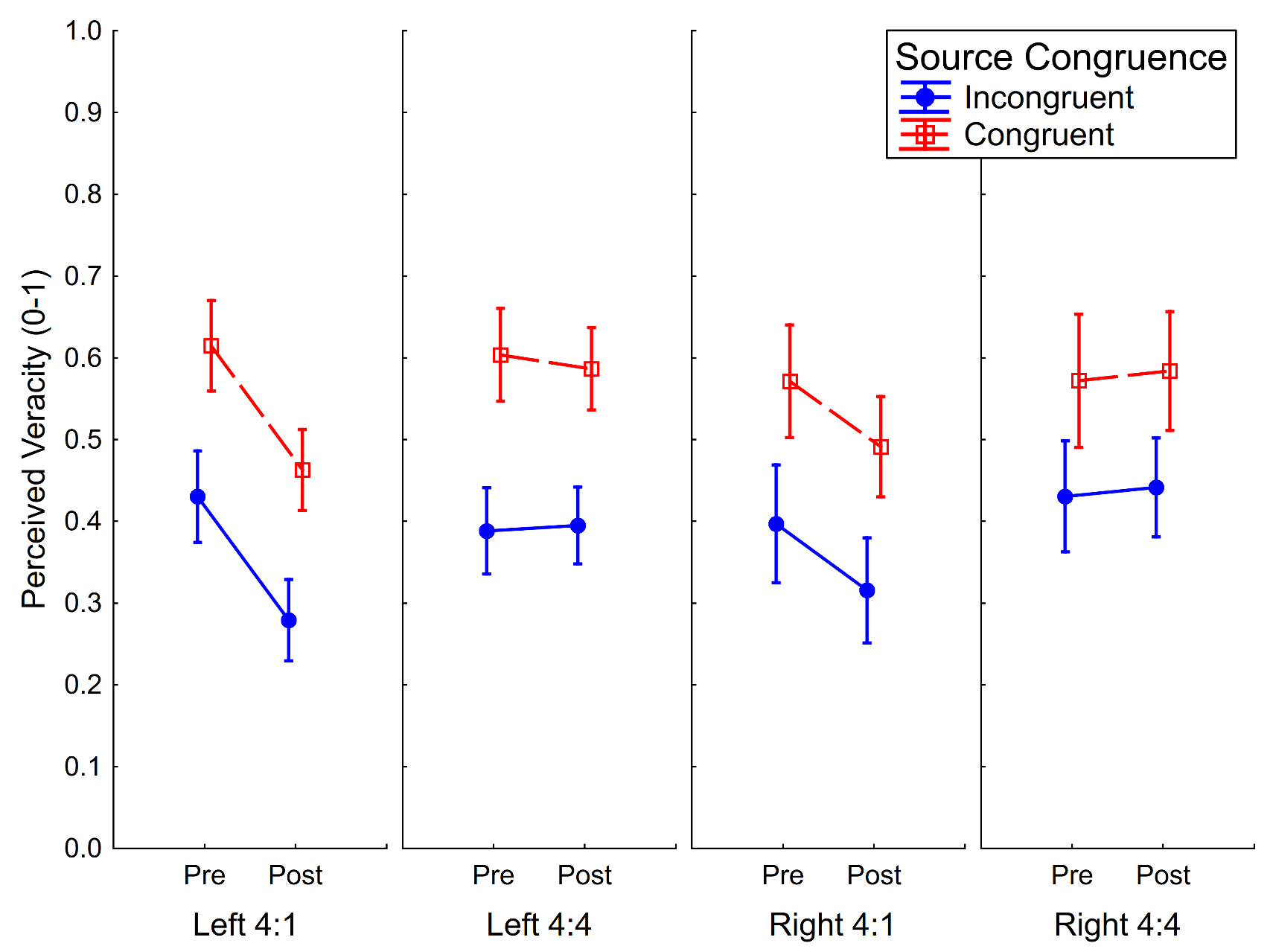
**
